# A Hybrid Active Noise Control System for the Attenuation of Road Noise Inside a Vehicle Cabin

**DOI:** 10.3390/s20247190

**Published:** 2020-12-15

**Authors:** Zibin Jia, Xu Zheng, Quan Zhou, Zhiyong Hao, Yi Qiu

**Affiliations:** College of Energy Engineering, Zhejiang University, Hangzhou 310027, China; zibin_jia@zju.edu.cn (Z.J.); zhouquan2010@zju.edu.cn (Q.Z.); haozy@zju.edu.cn (Z.H.); yiqiu@zju.edu.cn (Y.Q.)

**Keywords:** active noise control, hybrid control system, road noise, reduction performances, vehicle cabin

## Abstract

This paper proposed a local active control method for the reduction of road noise inside a vehicle cabin. A multichannel simplified hybrid active noise control (sHANC) system was first developed and applied to the rear left seat of a large sport utility vehicle (SUV). The attenuation capability of the sHANC system was investigated through simulations, using reference signals provided by accelerometers on the suspensions and bodywork of the vehicle and microphones on the floor of cabin, respectively. It was shown that compared to the traditional feedforward system, the sHANC system using either vibrational or acoustical reference signals can produce a significant suppression of the narrowband peak noise between 75 and 80 Hz, but the system lost the control capability in a range of 100–500 Hz when the acoustic signals were used as references. To reduce the practical implementation costs while maintaining excellent reduction performance, a modified simplified hybrid ANC (msHANC) system was further proposed, in which combined vibrational and acoustical signals were used as reference signals. The off-line analyses showed that four reference accelerometers can be substituted by ten microphones without compromising attenuation performance, with 3.7 dBA overall noise reduction being achieved. The effect of delays on the reduction performance of msHANC system was also investigated. The result showed that the msHANC system was more sensitive to the delays compared to the sHANC system if using only vibrational reference signals.

## 1. Introduction

Recently, the noise, vibration, and harshness (NVH) characteristics of vehicles have attracted great attention from vehicle manufacturers. Reducing the interior noise of a vehicle to improve driver and passenger comfort has become a pressing issue [1]. Since the 1980s, the application of active noise control (ANC) technology to reduce the interior noise of vehicles has been extensively investigated [2]. The first attempt at feedforward engine noise control was presented by Elliott et al. [3], and the developed engine noise control (ENC) system could reduce the sound pressure level at engine firing frequency between 10 and 15 dB. Due to the development of the digital signal processing (DSP), such a system has been implemented widely by a number of manufacturers [4,5]. Another application of active sound control for vehicle interior noise is road-type noise, which is a result of the interaction between the road surface and the vehicle tires. Compared to the ENC system, it is a great challenge to use a feedforward control strategy in the road noise control (RNC) system because of the broadband random nature of road excitation [6].

To achieve effective road noise control, the reference signals need to be highly correlated with the primary disturbance. However, in practice, due to the complex propagation path between structural and acoustic noise, it is difficult to obtain good coherence in the frequency band of interest. To overcome this limitation, Duan et al. [7] proposed a combined feedforward–feedback ANC system, which was formed by adding a feedback controller to the traditional feedforward ANC (tFANC) system; the simulation results showed that the hybrid control system can suppress the narrowband road noise component, which cannot be attenuated by feedforward structure operating alone, and it achieved a further 1 dBA of overall reduction. In their research, the internal model control (IMC) structure was used to build the feedback controller, which meant that the error signals and the secondary signals filtered by the estimation of secondary path were used to synthesize the reference signals for the feedback controller. Although this structure had great stability and robustness, as discussed by Padhi et al. [8], it had a heavy computational load by filtering the secondary signals, especially when the IMC structure was combined with the feedforward structure based on the Filter-x LMS (FxLMS) algorithm, which increased the cost of hardware. To reduce the burden for a real-time controller, Wu et al. [9] proposed a simplified feedback ANC system, in which the error signals were adapted directly as the reference signals for feedback structure and had the advantages of low computational load and ease implementation. The simulation and experimental results proved that the simplified system does not degrade the steady state performance significantly compared to the IMC feedback system. A simplified hybrid ANC (sHANC) system was also developed [10]; it showed great attenuation performance for uncorrelated disturbances which cannot be controlled by a feedforward structure. Using the traditional hybrid ANC (tHANC) system based on the IMC structure as a comparison, the sHANC system can improve the convergence rate and noise reduction performance by reducing the coupling relationship between the feedforward and feedback structures. However, these conclusions were drawn under the situation that the control system was aimed at a single-input single-output (SISO) problem and the primary path was accurately modeled. Moreover, the influence of the sound field characteristic within the enclosure was not considered in the specific simulations.

Another focus of implementing a road noise control system is the determination of the reference sensors. This is a common method to install accelerometers on the suspensions and bodywork of the vehicle to obtain the advance structural vibration signals caused by road excitation [11,12]. It has been shown that at least six accelerometers are needed to capture the primary components of road noise and achieve a reasonable noise reduction level [11]. Due to the high cost of accelerometers, this method shows limited commercial implementation. An attempt at reducing the cost of the control system was presented by Mohammad et al. [13], who mounted low-cost microphones on the floor of the vehicle cabin as reference sensors and proved that it had a similar attenuation capacity compared to the use of accelerometers. This system shows great application benefits, since a micro-electro-mechanical system (MEMS) microphone is much cheaper than an accelerometer nowadays. However, in their work, since the ANC controller was formulized in the frequency domain and lacked causality constraints, the effect of advanced time provided by microphone signals was not considered. Moreover, the microphone reference signals can be affected easily by the spatial variance of the sound field within the cabin, as mentioned by Cheer et al. [14], which limits the attenuation performance of the control system.

To make the sHANC system applicable in practical situations, in this paper, a multichannel sHANC system was developed to control the road noise inside a vehicle cabin. The attenuation performances of the aforementioned system, multichannel tFANC system, and tHANC system were all analyzed by using microphone or accelerometer reference signals to investigate the effects of different reference signals and control strategies. In addition, to reduce the implementation costs while maintaining the excellent noise attenuation performance of the sHANC system, a modified simplified hybrid ANC (msHANC) system using both microphone and accelerometer signals was also proposed in detail.

The remaining sections of this paper are organized as follows: Section 2 presents the formulation of the multichannel sHANC system in the time domain. In Section 3, the disturbance and reference signals measured inside a large sport utility vehicle (SUV) cabin are used to compare the attenuation performances of the multichannel sHANC system and other traditional systems. Then, the reduction performances of a modified sHANC system using both two types of reference signals are presented in Section 4. Finally, the conclusions of this work are provided in Section 5.

## 2. The Multichannel sHANC System

**Figure 1:**
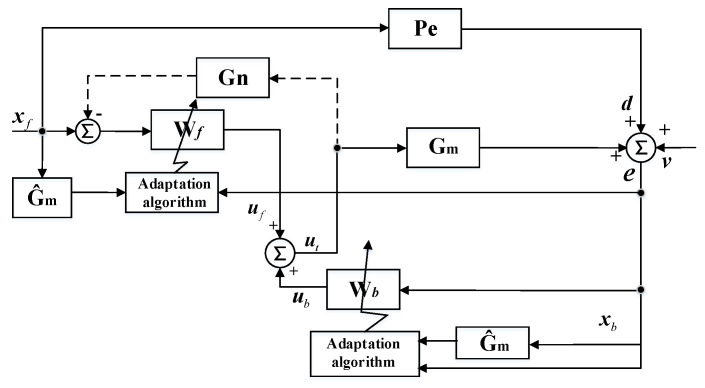
Block diagram of the MIMO simplified hybrid active noise control system, mainly including reference signals vector ***x****_f_*, error signals vector ***e***, two nominal plant responses matrices ***G****_m_* and ***G****_n_*, two control filter matrices ***W****_f_* and ***W****_b_*.

Road noise can be considered as stationary random broadband noise, and the control system should be constrained casually. In this case, the attenuation performance of the hybrid ANC system was investigated in the time domain [15]. A multiple-input multiple-output (MIMO) sHANC system was established and the block diagram of the system is shown in Figure 1.

In Figure 1, the feedforward structure uses a number of K reference sensors, and at nth sample time, the signals form a (K×1) vector, $x_{f}(n)={\left[x_{f1}(n),x_{f2}(n)\;\ldots x_{fK}(n)\right]}^{T}$. The MIMO control system aims to attenuate the road noise component around the passenger’s head. The number of error microphones used around the passenger’s head is defined as L. The (L×1) vector of error signals, $e(n)={\left[e_{1}(n),e_{2}(n)\;\ldots e_{L}(n)\right]}^{T}$, is given by the concatenation of L microphone signals, and e(n) can be expressed as:

(1) $$e(n)=d(n)+v(n)+G_{m}u_{t}^{\prime }(n)$$

where $d(n)={\left[d_{1}(n),d_{2}(n)\;\ldots d_{L}(n)\right]}^{T}$, which is produced by reference signal vector, $x_{f}(n)$, through the (L×K) matrix of transfer responses, $P_{e}$. The (L×1) vector of disturbances, $v(n)={\left[v_{1}(n),v_{2}(n)\;\ldots v_{L}(n)\right]}^{T}$, represents uncorrelated noise in the primary path. The $G_{m}$ is the matrix of nominal plant responses between the M secondary sources and L error microphones, and $u_{t}^{\prime }(n)$ represents the vector of current and past control signals for secondary sources.

The matrix of nominal plant responses, $G_{m}$, is defined as:

(2) $$G_{m}={\left[G_{1},G_{2}\;\ldots G_{L}\right]}^{T}$$

where $G_{l}={\left[G_{l1}^{T},G_{l2}^{T}\;\ldots G_{lM}^{T}\right]}^{T}$, and $G_{lm}$ is the vector of nominal plant responses between the mth secondary source and lth error microphone, which is modeled by an Ith order finite impulse response (FIR) filter and written as:

(3) $$G_{lm}={\left[G_{lm1},G_{lm2}\;\ldots G_{lm(I-1)}\right]}^{T}$$

In addition, the $u_{t}^{\prime }(n)$ is the output summation of control signal vector in feedforward structure and feedback structure and can be expressed as:

(4) $$u_{t}^{\prime }(n)=u_{f}^{\prime }(n)+u_{b}^{\prime }(n)$$

where $u_{f}^{\prime }(n)={\left[u_{f1}^{T},u_{f2}^{T}\;\ldots u_{fM}^{T}\right]}^{T}$ and $u_{b}^{\prime }(n)={\left[u_{b1}^{T},u_{b2}^{T}\;\ldots u_{bM}^{T}\right]}^{T}$. The $u_{fm}$ and $u_{bm}$ are signal vectors for mth secondary source of feedforward structure and feedback structure, respectively, which include the current and I−1 past control signals, which are defined as:

(5) $$u_{fm}={\left[u_{fm}(n),u_{fm}(n-1)\;\ldots u_{fm}(n-I-1)\right]}^{T}$$

(6) $$u_{bm}={\left[u_{bm}(n),u_{bm}(n-1)\;\ldots u_{bm}(n-I-1)\right]}^{T}$$

The control signal (M×1) vector of feedforward structure at nth sample time is defined as $u_{f}(n)={\left[u_{f1}(n),u_{f2}(n)\;\ldots u_{fM}(n)\right]}^{T}$, and the $u_{f}^{\prime }(n)$ can be derived from the buffer and shift operations of $u_{f}(n)$.

As shown in the block diagram, the $u_{f}(n)$ is obtained from the vector of reference signals $x_{f}(n)$ filtered through the control filter matrix $W_{f}$, which is given by:

(7) $$W_{f}={\left[w_{f1},w_{f2}\;\ldots w_{fM}\right]}^{T}$$

where $w_{fm}=\left[w_{fm1},w_{fm2}\;\ldots w_{fmK}\right]$, and $w_{fmk}$ is a vector of control filter between mth secondary source and kth refenence signal, which is a Jth order FIR filter:

(8) $$w_{fmk}=\left[w_{fmk1},w_{fmk2}\;\ldots w_{fmk(J-1)}\right]$$

Therefore, the $u_{f}(n)$ can be expressed as:

(9) $$u_{f}(n)=W_{f}x_{f}^{\prime }(n)$$

where $x_{f}^{\prime }(n)={\left[x_{f1}(n),x_{f1}(n-1)\;\ldots x_{f1}(n-J+1),x_{f2}(n)\;\ldots x_{fK}(n-J-1)\right]}^{T}$, which can be derived from the buffer and shift operations of $x_{f}(n)$.

For the purpose of adaptive control, the multichannel leaky FxLMS algorithm [16,17] is used to update the coefficients of the control filter matrix, $W_{f}$. In order to ensure the correct dimensions for matrix multiplication, the elements in matrix $W_{f}$ are rearranged, and the new (MKJ×1) matrix is defined as:

(10) $$W_{f}^{\prime }={\left[w_{f0}^{T},w_{f1}^{T}\;\ldots w_{f(J-1)}^{T}\right]}^{T}$$

where $w_{fi}={\left[w_{f11i},w_{f12i}\;\ldots w_{f1Ki},w_{f21i}\;\ldots w_{fMKi}\right]}^{T}$, and the updated equation of control filter matrix can be expressed as:

(11) $$W_{f}^{\prime }(n+1)=(1-\alpha_{f}\beta_{f})W_{f}^{\prime }(n)-\alpha_{f}{\hat{R}}_{f}{}^{T}(n)e(n)$$

where $\alpha_{f}$ is the step size of the feedforward control, $\beta_{f}$ is the leakage factor of feedforward control, which is used to improve the robustness of the algorithm, and ${\hat{R}}_{f}$ is the estimated filtered reference signal (L×MKJ) matrix, which can be represented as:

(12) $${\hat{R}}_{f}(n)=\left[\begin{array}{cccc} r_{f1}^{T}(n) & r_{f1}^{T}(n-1) & \cdots & r_{f1}^{T}(n-I+1)\\ r_{f2}^{T}(n) & r_{f2}^{T}(n-1) & & \vdots \\ \vdots & \vdots & & \vdots \\ r_{fL}^{T}(n) & r_{fL}^{T}(n-1) & \cdots & r_{fL}^{T}(n-I+1) \end{array}\right]$$

where $r_{fl}(n)={\left[r_{fl11}(n),r_{fl12}(n)\;\ldots r_{fl1K}(n),r_{fl21}(n)\;\ldots r_{flMK}(n)\right]}^{T}$, and $r_{flmk}(n)$ represents the kth reference signal which is filtered by the estimated plant response between the mth secondary source and lth error microphone, which is equal to:

(13) $$r_{flmk}\left(n\right)=\sum_{i=0}^{I-1}{\hat{G}}_{lmi}x_{fk}(n-i)$$

Then, considering the feedback structure in Figure 1, the reference vector of the feedback controller, $x_{b}(n)={\left[x_{b1}(n),x_{b2}(n)\;\ldots x_{bL}(n)\right]}^{T}$, is derived from the error signal vector directly, i.e.,

(14) $$x_{b}(n)=e(n)$$

Equation (14) shows that the feedforward structure will not have an effect on the feedback structure directly compared to the traditional hybrid ANC system, which will reduce the coupling between these two structures.

The control signal vector $u_{b}(n)$ is given by:

(15) $$u_{b}(n)=W_{b}x_{b}^{\prime }(n)$$

where $x_{b}^{\prime }(n)$ is derived from the buffer and shift operations of $x_{b}(n)$.

Similarly, the updated equation of the control filter matrix in the feedback structure can be expressed as:

(16) $$W_{b}^{\prime }(n+1)=(1-\alpha_{b}\beta_{b})W_{b}^{\prime }(n)-\alpha_{b}{\hat{R}}_{b}{}^{T}(n)e(n)$$

where $\alpha_{b}$ is the step size, $\beta_{b}$ is the leakage factor of feedback control, and $W_{b}^{\prime }$ and ${\hat{R}}_{b}{}^{T}(n)$ have a similar form to $W_{f}^{\prime }$ and ${\hat{R}}_{f}{}^{T}(n)$ in Equation (11), respectively. Similarly to the feedforward structure, the adaptive feedback controller directly uses the vector e(n) as the error signal input.

It should be noted that if the microphones are selected as reference sensors, a feedback path between control sources and references is needed to cancel the disturbances of the secondary sources on the reference signals, which is modeled by a (M×K) transfer response matrix $G_{n}$, as shown by the dotted line in Figure 1.

## 3. Active Noise Control of Road Noise Based on Multichannel sHANC System

### 3.1. Experimental Arrangement

**Figure 2:**
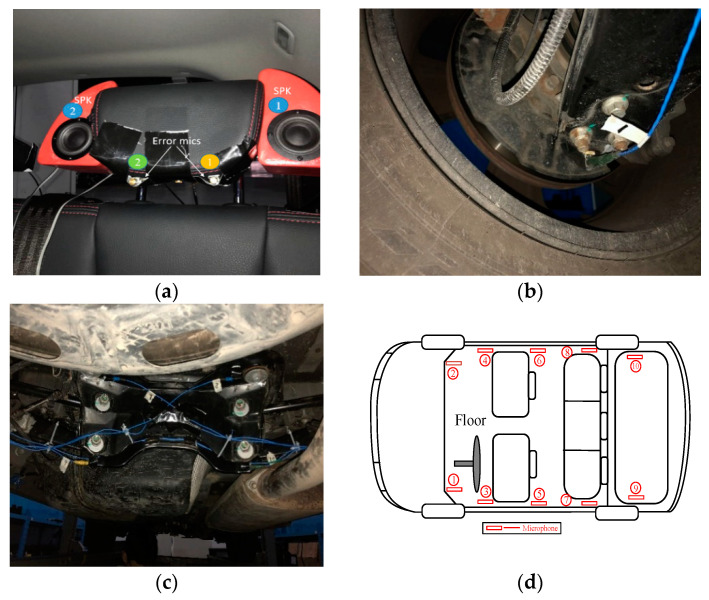
(**a**) The active headrest system at the rear left seat of the SUV containing two headrest speakers and two error microphones; (**b**) The installation location of accelerometer: the hubs of axle; (**c**) A few installation locations of accelerometers: the bushings of sub-frame, the joints between bodywork and sub-frame; (**d**) The schematic diagram of the numbered reference microphone placement.

Previous research has proven that, compared to the global control system, the local control system can extend the control frequency band while improving noise attenuation performance [15,18,19]. The local active noise control system was considered in this paper. To verify the potential performance of the simplified hybrid road noise control system as described in the previous section, the experiments were conducted in an SUV. Figure 2a shows the active headrest system at the rear left seat of the SUV, which contains two headrest speakers and two error microphones representing the position of the passenger’s ears. Before the experiments, the determination of the reference sensors for feedforward structure was considered. To compare the impact of different types of reference sensor on noise attenuation and minimize implementation costs, two placement schemes were arranged initially:

1. Vibration sensors: 12 triaxial accelerometers were numbered as references. They were mounted on the four suspension systems and tires, where the characteristics of road excitation transmitted through the structure can be captured, such as the hubs of the axle, as shown in Figure 2b, the bushings of the sub-frame, the joints between the bodywork and sub-frame, as shown in Figure 2c, and so on. Each accelerometer had three axes, so a total of 36 vibration signals were measured.
2. Microphone sensors: 10 microphones located on the floor of the vehicle cabin were numbered as references, and the exact position of each microphone is shown in Figure 2d.

The experiments were conducted when the SUV was driven at a constant speed of 80 km/h on a rough road, and the disturbance signals from error microphones and the reference signals of two defined schemes were obtained simultaneously.

### 3.2. Selection of Reference Signals

**Figure 3:**
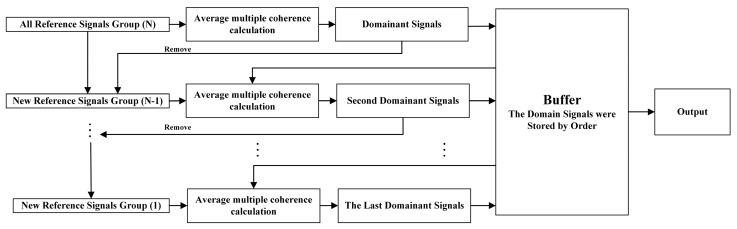
The flowchart of ranking the vibrational reference signals.

To ensure that the feedforward structure of the hybrid system can achieve good controlling performance, it is necessary to keep the reference signals correlated with disturbances. Theoretically, better coherence between the disturbances and reference signals can be achieved by using more reference sensors. Therefore, for a low-cost microphone placement scheme, firstly, all ten microphone inputs were used as reference signals. However, for the accelerometer placement scheme, due to the high cost of accelerometers, it is necessary to select the minimum number of sensors to maintain a relatively strong correlation with disturbances. Moreover, in actual implementation, triaxial accelerometers will be replaced by uniaxial accelerometers, so the goal became the selection of dominant vibration signals among all signals. As suggested by [7,11], the vibration signals can be ranked by calculating the average coherence between the vibration signals and each error signal in the control range, and the flowchart is shown in Figure 3. At the beginning, all the reference signals were calculated respectively to find the signal having the highest average coherence with the error signals, which was defined as the first dominant signal. Then, the reference signal was removed from all the reference signals and stored in the buffer. In the next iteration, along with reference signals in the buffer, the remaining reference signals were calculated again to find the second dominant signal based on the average multiple coherence function, which is defined as:

(17) $$J=\frac{1}{L}\sum_{l=1}^{L}\sum_{f_{lower}}^{f_{upper}}\gamma_{l}{}^{2}(f)$$

where $f_{lower}=20\;\mathrm{Hz}$, $f_{upper}=500\;\mathrm{Hz}$, representing the frequency band of interest. The multiple coherence function at the lth error microphone, $\gamma_{l}{}^{2}$, is defined as:

(18) $$\gamma_{l}{}^{2}=\frac{S_{xe_{l}}S_{xx}^{-1}S_{xe_{l}}^{H}}{S_{e_{l}e_{l}}}$$

Similarly, the second dominant signal was stored in the buffer by order. The above iteration continued until all the reference signals were ranked, and the corresponding ranking list of reference signals was output. The above rank operation was conducted based on the data when the SUV was driven at a constant speed of 80 km/h on a rough road. Through analysis, it was found that the vibration signals had a higher correlation with the error signals located in the rear suspension system of the SUV rather than the front suspension (for simplicity, the ranking list is not shown in this section).

**Figure 4:**
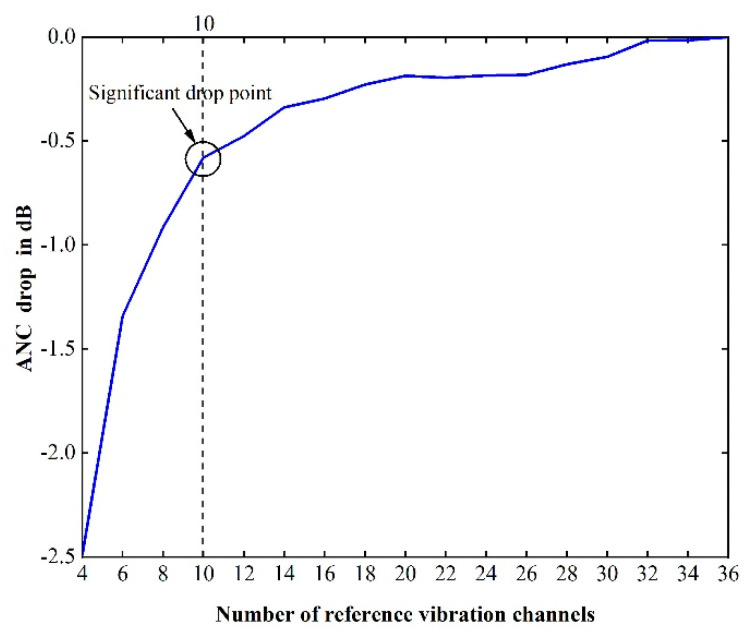
The predicted performance (overall reduction) of pure feedforward control system using different numbers of vibration channels.

Then, to remove some redundant accelerometers from pre-set positions, the simulations based on the feedforward structure (Figure 1) alone were operated multiple times with a decreasing number of references to observe how the attenuation performance dropped. According to the ranking lists, the lowest two reference channels were removed when a certain simulation was finished. For instance, the first simulation contained all 36 references, the second simulation contained the top 34 references, and the last simulation only contained the top four reference signals. The ANC performance (overall reduction) with the number of vibrational reference channels decreasing is shown in Figure 4. It can be seen that the ANC performance dropped significantly when the number of references was less than 10; hence, the top 10 vibrational reference signals were chosen for the following analysis.

**Figure 5:**
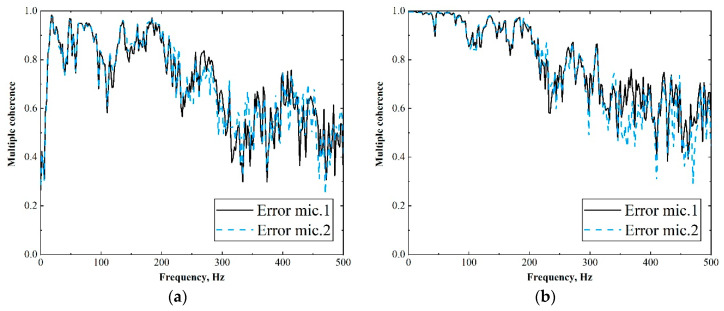
The multiple coherence between each of the error microphones and all selected vibrational or acoustical reference signals when the SUV was driven at a constant speed of 80 km/h on a rough road. (**a**) Ten vibrational reference signals; (**b**) Ten acoustical reference signals.

The multiple coherences between each error signal and selected reference signals are shown in Figure 5; when (a) ten vibration signals were used as reference signals, (b) ten acoustic signals were used as reference signals. From the results, it is interesting to observe that these two different types of reference signals were both highly correlated with each error signal until the frequency reached around 200 Hz, while at the higher frequencies, they lost the coherence, which limited the attenuation performance of the feedforward controller. These results also indicated that if the causality constraint of the control system was not considered, using acoustic signals as references could achieve a similar level of control effect as using vibration signals.

### 3.3. Plant Response Measurement

**Figure 6:**
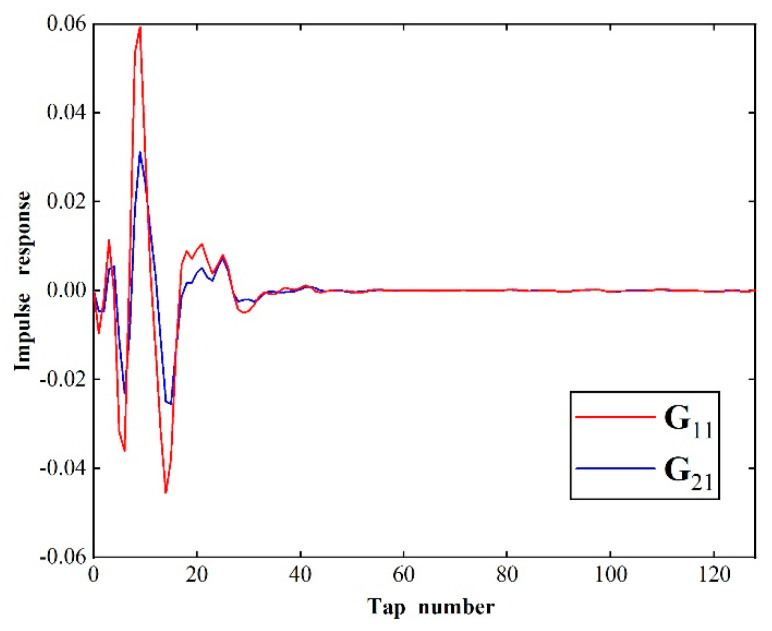
The impulse response (IRF) between left speaker 1 and two error microphones.

In order to implement the hybrid control system, the estimated plant responses matrix, ${\hat{G}}_{m}$, should be determined. In practice, the individual plant response between each headrest speaker and each microphone was modeled by an adaptive 128-tap FIR filter. Each headrest speaker was driven with a sweep signal independently to obtain the responses of the microphones. Along with the drive signals, these response signals were used to train the FIR filters. After the filters were converged, the coefficients of each FIR filter were considered as the estimated impulse response function (IRF). The IRFs between left speaker 1 and two error microphones are presented as an example in Figure 6. Moreover, the plots showed that the IRFs between the secondary speakers and the error microphones can be well modeled by 128-tap filters.

### 3.4. The Attenuation Performance of Multichannel sHANC System

**Figure 7:**
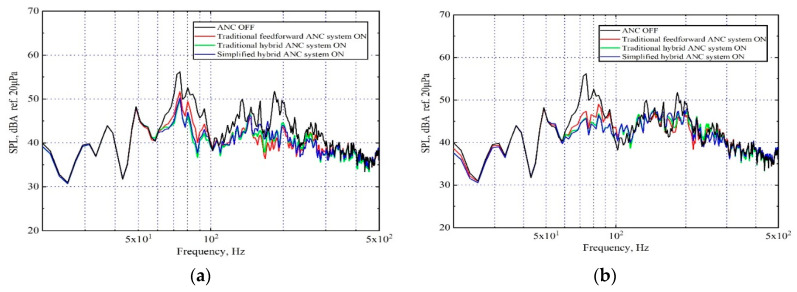
A-weighted averaged sound pressure level over two error microphones before and after control by applying different control systems, when the SUV was driven at a constant speed of 80 km/h on a rough road, (**a**) using ten vibrational reference signals; (**b**) using ten acoustical reference signals.

In this section, the off-line model is built to analyze the attenuation performance of the proposed MIMO sHANC system. As a comparison, the noise attenuation capability of tFANC and tHANC system are also presented. These systems were built using disturbance signals and the reference signals of two defined schemes, which were obtained simultaneously when the SUV was driven at 80 km/h on a rough road. In the simulations, to guarantee that the systems were effective and stable, the step size and leakage factor of the adaptive feedforward (feedback) controller were chosen by trial and error empirically. Figure 7 compares the effects of different reference signals and control systems on noise reduction performance. Figure 7a shows the performance of the sHANC system compared to two traditional ANC systems using vibration reference signals. From the sound pressure spectrum, it can be seen that when vibration signals were used as references, more significant suppression of the narrow peak from 70 to 85 Hz could be achieved by using the sHANC and tHANC systems, compared to tFANC system. This phenomenon indicated that the 70–85 Hz uncorrelated narrow disturbance cannot be controlled by the pure feedforward controller, while it can be reduced by adding the feedback structure. For the broadband range 100–500 Hz, it can be observed that all the control systems showed a relatively good reduction capability, though better reduction performance was limited because of the poor coherence between the reference signals and error signals at higher frequencies, as shown in Figure 5a. These results also showed that the sHANC system can achieve similar noise reduction to the tHANC system. Compared to the tFANC system, the sHANC system not only achieved 3 dBA more attenuation in the narrow band of 70–85 Hz but also achieved a further 0.3 dBA of the overall reduction.

The same reduction performances of control systems using all acoustical reference signals are shown in Figure 7b. An interesting observation was that all control systems were efficient in noise reduction of the 70–85 Hz narrow peak, while the performances of the sHANC and tHANC system improved slightly. It can be inferred that the reference microphones on the cabin floor can detect more information about the narrow peak disturbance, and this conclusion can also be verified by the coherence results in Figure 5b. However, for the 100–500 Hz broadband range, the tHANC system made only a slight difference compared to the system when acceleration signals were used as references (Figure 7a). This may be caused by the unsatisfied causality of the feedforward controller, i.e., the microphone signals cannot provide enough advanced time. Moreover, within this frequency band, the other two hybrid systems seemed to demonstrate no improvement in noise reduction. This was because the delays in the plant responses limit the performance and achievable attenuation of the feedback controller, as discussed in [6,13]. Therefore, based on the above findings, to reduce the implementation costs by substituting a few accelerometers with microphones while maintaining the good noise attenuation performance of the sHANC system, the combination of acceleration and acoustic signals as reference signals can be considered.

## 4. Optimization of Attenuation Performance for Road Noise Control

### 4.1. Proposed Modified System

**Figure 8:**
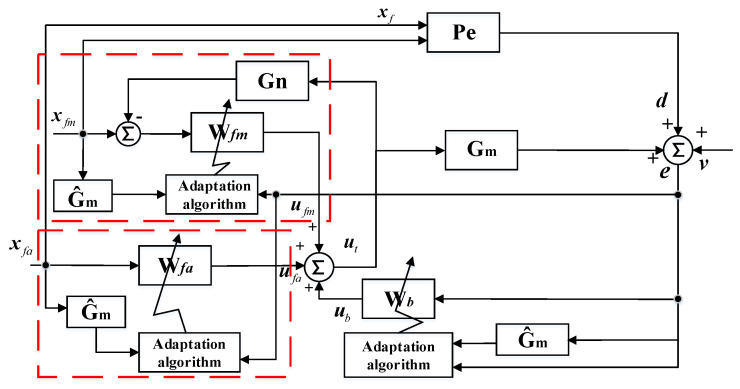
Block diagram of the MIMO modified simplified hybrid active noise control system (the two feedforward structures are indicated by the dashed rectangles), mainly including two reference signals vectors ***x****_fm_* and ***x****_fa_*, error signals vector ***e***, nominal plant responses matrices ***G****_m_* and ***G****_n_*, three control filter matrices ***W****_fm_*, ***W****_fa_* and ***W****_b_*.

Due to the difference in the property of the vibration signals and acoustic signals, if these two types of signal are composed directly into the reference vector, $x_{f}$, as shown in Figure 1, it will distort the input signals and further bias the control effect of the controller. Therefore, these two parts of reference signals need to be input independently. To overcome this phenomenon, a modified MIMO simplified hybrid ANC (msHANC) system was proposed, and the block diagram is shown in Figure 8. Compared to the sHANC system, the msHANC system employs two feedforward structures containing filter matrix $W_{fm}$ and $W_{fa}$, respectively. The former is called a microphone-based feedforward structure, which is mainly devoted to controlling the 70–85 Hz narrow peak response using microphone signals. The latter is an accelerometer-based feedforward structure, which uses vibration signals to control the residual noise spectrum between 100 and 500 Hz that cannot be controlled by the former structure. The $K_{m}\times 1$ vector of microphone signals and the $\left(K_{a}\times 1\right)$ vector of vibration signals give the full reference signal vector, $x_{f}={\left[x_{fm}\;x_{fa}\right]}^{T}$, and the correlated disturbance d is produced by $x_{f}$ via the matrix of transfer responses, $P_{e}$. As mentioned in the previous section, it will be a feedback path $G_{n}$ between control sources and reference microphones, and it is assumed to be modeled perfectly in this paper, so that the $x_{fm}$ obtained from experiments can be considered as the “true” reference acoustic signals.

In the msHANC system, the control signal vector $u_{t}^{\prime }(n)$ can be expressed as:

(19) $$u_{t}^{\prime }(n)=u_{fm}^{\prime }(n)+u_{fa}^{\prime }(n)+u_{b}^{\prime }(n)$$

where $u_{fm}^{\prime }(n)$, $u_{fa}^{\prime }(n)$, and $u_{b}^{\prime }(n)$ are control signal vectors derived from two feedforward structures and one feedback structure, respectively. The signal generation in the msHANC system, including the updated equation of three control filter matrixes, is similar to the sHANC system; for simplicity, it is not re-explained in this section.

### 4.2. Performance Assessment of the msHANC System

**Figure 9:**
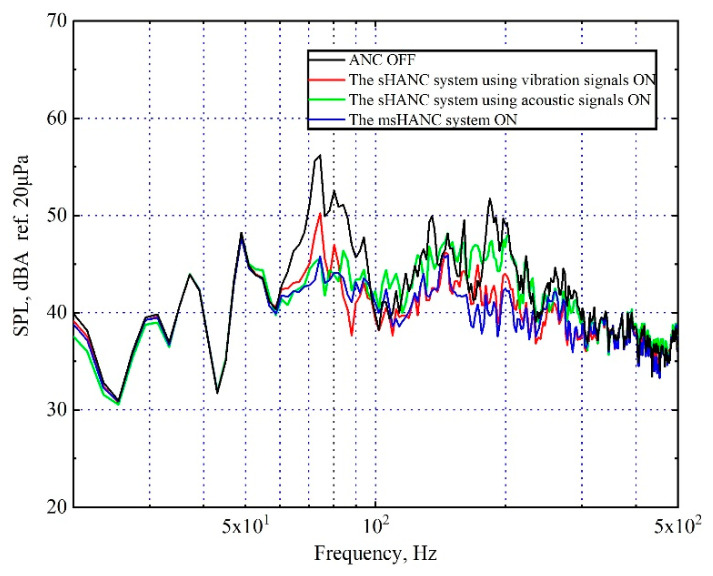
A-weighted averaged sound pressure level over two error microphones before and after control by sHANC system using two types of reference signals independently, the proposed msHANC system using both reference signals, when the SUV was driven at a constant speed of 80 km/h on a rough road.

The spectrum of the averaged sound pressure level measured at two error microphones after being controlled by the proposed msHANC system (using ten vibrational reference signals and ten acoustical reference signals) is shown in Figure 9, and the results controlled by the sHANC system using two types of reference signals independently are re-plotted for comparison. From these plots, it can be seen that the proposed msHANC system combined the advantages of using vibration reference signals and acoustic reference signals alone; that is, it can achieve both high levels of narrowband attenuation between 70 and 85 Hz and broadband attenuation between 100 and 500 Hz. The msHANC system achieved a maximum overall reduction of 4.1 dBA, which is 0.5 dBA more overall reduction compared to the sHANC using vibration reference signals alone.

**Figure 10:**
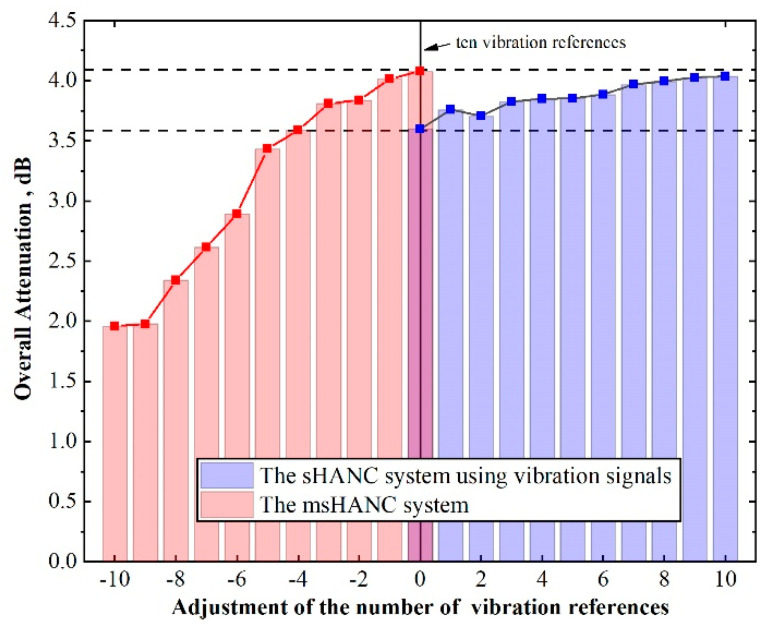
The predicted performance (overall reduction) of two control systems with the increasing/decreasing of the number of vibration references (the two control systems both contain ten vibration signals at the beginning).

As mentioned in the previous section, to reduce the implementation costs, a few accelerometers were supposed to remove from selected reference sensors. Thus, it is interesting to predict the noise attenuation performance of the msHANC system as the number of vibration references decreases. To demonstrate the effectiveness of the proposed system, the performance of the sHANC system using vibration reference signals was also predicted as the number of vibration references increased. The two control systems both contained ten vibration signals at the first simulation, and the results are shown in Figure 10. It is worth highlighting that the selection of vibration references in each adjustment depended on the ranking list obtained previously.

From the results in Figure 10, it can be seen that when ten microphone signals and the top ten vibration signals were integrated into the full reference signal vector $x_{f}$ for the msHANC system (Figure 8), the system could achieve slightly more noise reduction compared with the sHANC system using the top twenty vibration signals. This meant that if the number of reference signal channels was the same, ten accelerometers could be replaced by ten microphones among twenty accelerometers to obtain similar attenuation performance. Moreover, it can be seen that when ten microphone signals and the top six vibration signals were integrated into the full reference signal vector, the msHANC system achieved the same overall noise reduction compared with the sHANC system using the top ten vibration signals, which obtained an overall reduction of approximately 3.7 dBA. In this case, this meant that four reference accelerometers among ten essential accelerometers could be substituted by ten microphones while maintaining the same attenuation performance. Based on the comparison, it can be concluded that the msHANC system using both microphone signals and vibration reference signals can improve the attenuation performance significantly. It also provided the potential to further reduce the required number of accelerometers by using a few low-cost microphones, such as MEMS microphones.

### 4.3. Effect of Time Delays on Performance

**Figure 11:**
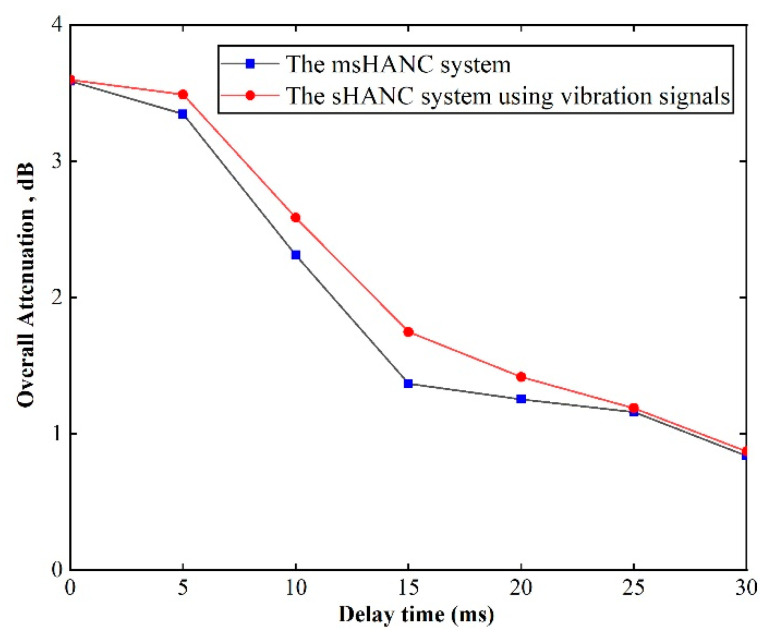
The predicted overall reduction performance of two control systems when different delays are added into reference signals.

For the practical implementation of the msHANC system, the delays of the real-time system need to be considered, which mainly consist of two types. As discussed [15,20], one type is caused by electronic, such as analog-to-digital/digital-to-analog (AD/DA) conversions, antialiasing/smoothing filters, and reference sensors, and it has been included in the obtained secondary transfer responses and reference signals. The other type of delay is caused by digital signal processing, especially in the multichannel signal convolution operations. To investigate the effect of processing delays on the msHANC system, a few additional delays were added into the reference signals, and the performance of the msHANC system using microphone signals and the top six vibration signals, which were verified to have the same attenuation capacity as the sHANC system using the top ten vibration signals, was analyzed off-line, as shown in Figure 11. The performance of the sHANC system using vibration signals was also demonstrated for comparison. From these results, it can be seen that the msHANC system was more sensitive to the delays, which may be due to the microphone signals used in the msHANC system being collected from the floor inside the cabin, while the sHANC system used only the vibration signals obtained from suspensions or wheels, which lost the causal constraint more easily as delays increased. In this case, the overall reduction of the msHANC system decreased by approximately 1 dB when additional delays exceeded 10 ms. Moreover, it was also shown that when the additional delays exceeded 20 ms, both control systems seemed to lose the capacity to control the disturbance and the overall reduction was reduced to 1 dBA. Therefore, in the practical implementation, it is recommended to choose the optimal controller and hardware to keep the processing time to less than 10 ms to maintain the system’s effectiveness.

## 5. Conclusions

A local active noise control system can achieve effective control of broadband random road noise when the reference signals are highly correlated with the primary disturbance. However, due to the complex propagation path between structural and acoustic noise, it is not practical to obtain great coherence in the frequency band of interest. To overcome this limitation, in this paper, the potential of applying a multichannel simplified hybrid active noise control (sHANC) system to the rear left seat of a large SUV has been investigated. Furthermore, to reduce the practical implementation costs while maintaining great reduction performance, a modified simplified hybrid control (msHANC) system has been proposed firstly. The main conclusions are as follows:

- The attenuation performance of the multichannel sHANC system was off-line analyzed, using the disturbance signals along with two types of reference signals: (a) ten vibration reference signals provided by accelerometers on the suspensions and bodywork of the vehicle, which were chosen out of 36 options; (b) ten acoustical reference signals provided by microphones on the floor of the cabin. It has been shown that, compared to the traditional feedforward system, the sHANC system using two types of reference signals can produce a significant suppression of the narrowband peak noise between 75 and 80 Hz, but the sHANC system lost the control capability in a broadband range of 100–500 Hz by using acoustical reference signals. It was also shown that compared to traditional hybrid control systems, the sHANC system can achieve similar noise reduction.
- The msHANC system using both vibrational reference signals and acoustical reference signals was proposed firstly. The system cleverly combined the benefits of using two different types of reference signals. The results showed that four reference accelerometers can be substituted by ten microphones without damaging the control performance, achieving around 3.7 dBA overall reduction. The system provided the potential to reduce the required number of accelerometers by using a few low-cost microphones, such as micro-electro-mechanical system (MEMS) microphones, which can further reduce the practical implementation costs.
- The effect of time delays on the reduction performance of the proposed systems was investigated. It has been shown that the msHANC was more sensitive to the delays compared to the sHANC system using only vibrational reference signals. When the additional delays exceeded 20 ms, the msHANC system lost the capacity to control the disturbance.
